# Unraveling the redox code to improve physiological research in human health and disease

**DOI:** 10.14814/phy2.70105

**Published:** 2024-10-31

**Authors:** Josh Thorley

**Affiliations:** ^1^ School of Sport, Exercise, and Health Sciences Loughborough University Loughborough UK

**Keywords:** cysteine, hydrogen peroxide, redox signaling, ROS

## Abstract

Redox reactions, involving electron transfer, are critical to human physiology. However, progress in understanding redox metabolism is hindered by flawed analytical methods. This review highlights emerging techniques that promise to revolutionize redox research, enhancing our comprehension of human health and disease. Oxygen, vital for aerobic metabolism, also produces reactive oxygen species (ROS), such as superoxide and hydrogen peroxide. While historically seen as harmful, ROS at low concentrations are now recognized as key regulators of cell signaling. A balance between ROS and antioxidants, known as redox balance, is crucial, and deviations can lead to oxidative stress. Recent studies have distinguished beneficial “oxidative eustress” from harmful “oxidative distress.” New techniques, such as advanced mass spectrometry and high‐throughput immunoassays, offer improved accuracy in measuring redox states and oxidative damage. These advancements are pivotal for understanding redox signaling, cysteine oxidation, and their implications for disease. Looking ahead, the development of precision redox medicine could lead to better treatments for oxidative stress‐related diseases and foster interventions promoting health.

## Graphical abstract

Redox biology is fundamental to human physiology; however, analytical techniques have lacked in previous years. Fortunately, a new era of techniques had arrived, enabling redox metabolism to be analyzed in more detail than ever.

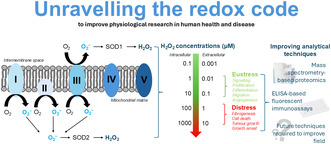

Oxidation–reduction (redox) reactions, defined by the transfer of electrons between molecules, are fundamental to human physiology; a sentiment underlined by the renowned biochemist Albert Szent‐Györgyi who famously said that “*life is nothing but an electron looking for a place to rest*.” Indeed, redox metabolism plays an essential, multifaceted role in regulating a plethora of cellular processes (Sies & Jones, 2020); however, progress in unraveling the complexities of redox metabolism is hampered by inappropriate analytical methods that are methodologically and conceptually flawed (Murphy et al., 2022). Fortunately, advancements in techniques aimed at quantitatively examining redox status offer promising opportunities to accelerate discoveries in human physiology. In this brief review, by introducing a set of innovative analytical techniques, I aim to convince you that advances in the field of redox metabolism over the next decade will be at the forefront of defining novel mechanisms that improve our understanding of human health and disease.

At the most fundamental level of human physiology, oxygen (O_2_) is crucial for sustaining life by perpetrating aerobic metabolism. This familiar facet of O_2_ enshrouds its other, perhaps lesser‐known fate; indeed, a small percentage of inhaled O_2_ is transformed into reactive oxygen species (ROS), a collective term to describe unstable derivatives of O_2_ produced across multiple cellular compartments (Forrester et al., 2018). Herein, it is important to clarify that ROS is used to describe diverse reactive and non‐reactive molecules, including reactive nitrogen and sulfur species; however, the term ROS here will only describe O_2_ centered reactants. The most physiologically relevant ROS formations include superoxide (O_2_^−^) and hydrogen peroxide (H_2_O_2_), which are capable of oxidizing lipids, nucleic acids, and proteins—actions representative of their reactive namesake (Jones, 2006).

Seminal investigations were quick to demonize ROS as injurious byproducts that irreversibly damage biomolecules which, if unaddressed, contribute to increased disease risk (Sies, 1985). Fortunately, ROS can be maintained at low intra‐ and extracellular homeostatic concentrations through the intricacies of powerful antioxidant defense systems which sense and retaliate to deviations in the steady‐state set point of ROS across cellular compartments (Pisoschi & Pop, 2015). This fine‐tuned equilibrium between ROS and antioxidants is routinely described as *redox balance,* and deviations in this balance were first described by Hans Seis in 1985 as a state of *oxidative stress*, which alludes to an imbalance in ROS and antioxidants, in favor of the former, leading to *oxidative damage* of proximal biomolecules (Sies, 1985). A series of investigations followed which challenged this myopic perspective, notably that ROS—foremost H_2_O_2_—when observable at low steady‐state intracellular concentrations (1–10 nM), are fundamental coordinators of cell signaling events (Sies, 2017). Two subcategories of oxidative stress therefore presently exist; these are *oxidative eustress*, which defines the beneficial adaptations regulated by low ROS exposure (good stress) and *oxidative distress*, which denotes the damaging effects to biomolecules that arise after high ROS exposure (bad stress) (Sies, 2017).

Owing to its distinct chemical properties, selective reactivity toward biological targets, diffusion properties, and abundance of enzymes maintaining its concentration, H_2_O_2_ is considered an important signaling molecule that has a critical regulatory role in metabolism, transcription regulation, cytoskeletal reorganization, and cell replication. As a classic example, H_2_O_2_ signaling modulates the activity of nuclear factor erythroid 2‐related factor 2, a transcription factor responsible for regulating the expression of >200 proteins with antioxidative, anti‐inflammatory, and detoxifying properties (Vomund et al., 2017). Such ROS‐induced signaling is defined as *redox signaling* and is predominantly driven by reversible oxidation of cysteine (Cys) thiol residues on target proteins (Paulsen & Carroll, 2013).

Indeed, the sulfur‐based chemical structure endowing Cys means that upon direct thiol oxidation by ROS or, alternatively, indirect modification by secondary relay signals (i.e., peroxiredoxins previously oxidized by ROS), reduced Cys can undergo various oxidative post‐translational modifications (Winterbourn & Hampton, 2008). For instance, under states of oxidative eustress, Cys are oxidized to form low oxidation state sulfenic acids and disulfides; highly reversible species that can be reduced by glutathione and thioredoxin antioxidants, thereby acting as molecular redox switches that initiate changes to various cell properties (Sies et al., 2024). Conversely, states of oxidative distress can overwhelm antioxidant defense systems, evoking hyperoxidation of Cys thiols that produce irreversible, higher oxidation products such as sulfinic and sulfonic acid (Sies et al., 2024). Formation of these hyperoxidated species typically marks the occurrence of oxidative damage, with excess formation of these species associated with disease pathology, including cancer and Alzheimer's (Sabharwal & Schumacker, 2014).

Measuring dynamic changes in the redox landscape is therefore highly valuable in offering insight into the potential eustress or distress responses coordinated by ROS. Traditionally, assessing redox status in vivo required an array of commercial assays and probes designed to quantify changes in biomarkers thought to indicate altered redox balance in isolated tissues (Halliwell & Whiteman, 2004). These biomarkers have commonly included:

1. Markers of oxidative damage: 8‐hydroxydeoxyguanosine (DNA oxidation), 4‐hydroxynonenal (lipid peroxidation), protein carbonyls (protein oxidation), 8‐isoprostanes (lipid peroxidation).
2. Markers of ROS production: Fluorescent probes like dihydroethidium and MitoSOX are commonly used to estimate cellular and mitochondrial O_2_^−^ production, respectively.
3. Markers of antioxidant activity: Antioxidant activity assays which detect the activity of a given antioxidant (i.e., glutathione peroxidase is detected by measuring the oxidation rate of NADPH to NADP^+^); total antioxidant capacity (TAC) assays, which determine the concentration of antioxidants within samples).

However, these techniques face several limitations. For instance, antibodies used in some ELISAs specific to oxidative damage biomarkers can lack specificity and sensitivity, resulting in high cross‐reactivity and background interference. Furthermore, antioxidant activity assays do not directly measure the antioxidant itself; rather, the product of the enzymatic reaction, which can be produced independently of the antioxidant being studied. TAC assays are also discouraged, notably because they do not measure all possible antioxidants, including superoxide dismutase and glutathione peroxidase enzymes (Pompella et al., 2014). Nonetheless, many of these kits are affordable, require minimal technical expertise, and are high throughput. These assays can also be “hacked,” improving their validity, and have been successful in identifying oxidative damage in disease states; thus, they should still be used, but with caution. Directly measuring ROS has been a particularly challenging task; commonly used fluorescent probes, like dihydroethidium, produce ethidium and 2‐hydroxyethidium—non‐specific oxidation products that obfuscate interpretation. Despite recent advancements in techniques for measuring H_2_O_2_ in tissues, such as MitoP to MitoB probes (Cairns et al., 2021), unavoidable changes in O_2_ exposure to isolated tissue lead to artificial increases in O_2_^−^. Furthermore, many lysis buffers required for sample processing generate artificial H_2_O_2_. These challenges, among others, make accurately measuring ROS in vivo a near‐impossible task and perhaps should be avoided for now.

These limitations have likely discouraged researchers from exploring the redox landscape; however, in recognition of the central role of oxidative distress in pathology and eustress in biological adaptation, there has been a significant effort to improve analytical techniques in this field. Regarding oxidative damage biomarkers, optimization of mass spectrometry techniques has revolutionized the measurement of oxidized proteins. For example, ultra‐performance LC–MS/MS analysis of 8‐hydroxydeoxyguanosine can overcome many of the shortcomings presented during ELISAs, including improving the sensitivity and specificity of analysis (Wu et al., 2017).

Recently, new techniques that focus on quantifying the versatility of Cys redox state in vivo have also been developed. At the turn of the decade, Xiao and colleagues (Xiao et al., 2020) introduced a comprehensive and quantitative mapping system to measure redox signaling activities across the Cys proteome in various tissues using mass spectrometry‐based proteomics. Despite only currently being validated in mouse models, this cutting‐edge technique utilizes a mechanism by which cell‐ and tissue‐specific reversible Cys oxidation is assessed by Cys‐reactive phosphate tag technology. Using this technology led to the evaluation of protein Cys oxidation patterns in young and aged mice, revealing significant losses in redox networks governed by Cys oxidation in aged mice, providing insight into how aging impacts redox metabolism and possibly redox dysregulations that underlie age‐related diseases.

High‐throughput, cost‐effective ELISA‐based fluorescent immunoassays have also been developed and validated, which offer a more accessible means of assessing target‐specific Cys oxidation in human tissues on a microplate; these are the antibody‐linked oxi‐state assay (ALISA) and the RedoxiFluor assay (Noble et al., 2021; Tuncay et al., 2022). ALISA measures thiol redox state by employing a thiol‐reactive fluorescent‐conjugated maleimide reporter to label reversibly oxidized thiols and a capture antibody to bind the target protein. A recent study by Muggeridge and colleagues used the ALISA to quantify human erythrocyte PP2A thiol oxidation after a bout of maximal exercise, discovering that PP2A reversible thiol oxidation decreased post‐exercise (Muggeridge et al., 2022). The RedoxiFluor assay, developed by Tuncay and colleagues, similarly utilizes fluorescent‐conjugated reporters to label reduced and reversibly oxidized thiols. A target‐specific capture antibody immobilized in a microplate is then used to bind the target from a sample and can subsequently be quantified in relative percentage and molar terms.

Measuring Cys oxidation state, in collaboration with the performance of optimized assays to detect oxidative damage biomarkers, could therefore be a valuable strategy in understanding the mechanistic underpinning of redox signaling and subsequent changes to redox metabolism toward various conditions or interventions. In unraveling these complexities, I believe that future advances in this field will help expand physiological research by allowing for the development of precision redox medicine, enabling more effective treatments to prevent disease characterized by oxidative distress, as well as uncovering interventions that promote health and wellbeing.

## FUNDING INFORMATION

No funding supported the creation of this review article.

## CONFLICT OF INTEREST STATEMENT

The author declares no conflicts of interest.

## ETHICS STATEMENT

This article was prepared solely by Dr. Josh Thorley under the guidelines stated as part of the ECR short review competition.
